# Can we predict the future of patients with liver cirrhosis using volumetrics?

**DOI:** 10.1002/ueg2.12308

**Published:** 2022-09-12

**Authors:** Marten A. Lantinga, Laurens A. van Kleef

**Affiliations:** ^1^ Department of Gastroenterology and Hepatology, Amsterdam Gastroenterology and Metabolism University Medical Centers Amsterdam Amsterdam The Netherlands; ^2^ Department of Gastroenterology and Hepatology Erasmus MC University Medical Center Rotterdam The Netherlands

In the article on computed tomography (CT) volumetrics in liver cirrhosis Romero‐Cristóbal et al. investigated the relationship between changes in liver‐spleen volume and features of cirrhosis in patients with compensated or decompensated liver cirrhosis who either underwent liver transplantation or partial hepatectomy for hepatocellular carcinoma (HCC).^1^

The authors showed in this Spanish cross‐sectional single center study that changes in liver volume, liver segmental volume ratio and liver‐spleen volume ratio evaluated by CT reflected the course of disease progression through the different stages of liver cirrhosis. Specific changes in volumetrics appeared to be related to compensated cirrhosis, compensated cirrhosis with development of portal hypertension and decompensated liver cirrhosis. Interestingly, these changes were independent of the predictive value of histological level of liver fibrosis. The authors concluded that the observed changes in liver and spleen volumes correlate with the different clinical stages in the course of liver disease progression. In turn, this would suggest that CT volumetrics of liver and spleen could be a readily available non‐invasive tool to provide prognostic information in cirrhotic patients.

These findings are of utmost importance, as one of the most essential challenges clinicians face in guiding treatment of patients with liver cirrhosis is stratifying the risk of disease progression, the development of decompensating events or even outcomes in hepatocellular carcinoma.^2^, ^3^ An easy to use non‐invasive tool could provide clinicians with a highly anticipated instrument to identify those patients at risk and introduce targeted interventions.^4^ These volumetric indices, although promising, require further validation with a focus on useful clinical thresholds for being at risk of disease progression. These studies should not be limited to patients that underwent liver transplants or partial hepatectomy but be performed across the entire spectrum of advanced liver disease.

Central, and current gold standard, in the risk stratification of patients with liver cirrhosis is estimating hepatic venous pressure gradient (HVPG) with HVPG measurement.^3^ As an alternative to this invasive procedure, transient elastography of liver and spleen have emerged as a non‐invasive alternative.^5^, ^6^ Although, it remains to be elucidated which cut‐offs are predictive of future risk of decompensation. Moreover, transient elastography of spleen and liver are, by definition, influenced by hemodynamic changes, caused by for example, atrial fibrillation.^7^

The strategy of volumetrics would provide an alternative to transient elastography in risk stratification in cirrhotic patients, without the risk of hemodynamic alternations being responsible for negatively influencing its predictive capacity. These techniques could however be implemented complementary and the overall diagnostic accuracy might improve using these techniques simultaneously.

Interestingly, volumetrics appear to be able to classify the stage of liver disease independent of the level of liver fibrosis as there was no association observed between volumetrics and intensity of fibrosis in this cohort of cirrhotic patients that underwent liver transplant or partial hepatectomy. This would suggest that fibrosis and parenchymal extinction are two independent factors affecting disease course, which in turn could explain that some patients with less severe fibrosis (but still F4) with intense parenchymal extinction may present a decrease of liver volume. As a result of the effect of volumetric changes independent of the predictive value of fibrosis stage, patients at risk of decompensation could potentially be more effectively identified additively using volumetrics.

Future study prospectively comparing different non‐invasive techniques should determine which tools should be used when in order to provide a patient‐tailored risk assessment.

## Data Availability

Data sharing is not applicable to this article as no new data were created or analyzed in this study.
